# Identification and characterization of circular RNA in the model of autism spectrum disorder from PM_2.5_ exposure

**DOI:** 10.3389/fgene.2023.970465

**Published:** 2023-05-09

**Authors:** Xiaoqian Xie, Kang Li, Xiaotian Liang, Lei Tian, Bencheng Lin, Jun Yan, Yue Shi, Xiaohua Liu, Zhuge Xi

**Affiliations:** ^1^ Tianjin Institute of Environmental and Operational Medicine, Tianjin, China; ^2^ Binzhou Medical University, Yantai, Shandong, China

**Keywords:** PM_2.5_, circRNA, autism spectrum disorder (ASD), transcriptome sequencing, RNA-seq

## Abstract

PM_2.5_ induces a series of effects on neurological disorders, including autism spectrum disorder (ASD), however, the mechanism is not completely clear yet. Circular RNAs (circRNAs) are a class of closed-loop structures that can be stably expressed *in vivo*. In our experiments, rats exposed to PM_2.5_ exhibited autism-like phenotypes, such as anxiety, and memory loss. To explore the etiology, we performed transcriptome sequencing and found significant differences in the expression of circRNA. A total of 7770 circRNAs were identified between the control and experimental groups, 18 of which were differentially expressed, we selected ten circRNAs and performed qRT-PCR and Sanger sequencing to validate them. By GO and KEGG enrichment analysis, we found differentially expressed circRNAs that were mainly enriched in processes related to placental development and reproduction. Finally, using bioinformatics, we predicted miRNAs and mRNAs that circ-Mbd5 and circ-Ash1l might regulate and constructed circRNA-miRNA-mRNA networks involving genes associated with ASD, suggesting that circRNAs might regulate the occurrence of ASD.

## 1 Introduction

Air pollution is a global concern owing to its impact on air quality and public health. Exposure to ambient air pollution could increase morbidity and mortality and is the leading contributor to the global burden of disease (Cohen et al., 2017). PM_2.5_, the major air pollutant, is defined as particulate matter with an aerodynamic diameter less than or equal to 2.5 μm and is also called fine particulate matter. The toxic and adverse effects of PM_2.5_ on human health have been confirmed (Hayes et al., 2020; Kim et al., 2020). Exposure to PM_2.5_ has a strong association with pulmonary and cardiovascular diseases (Liu et al., 2019). Recently, studies have also reported that PM_2.5_ is also associated with a series of neurological disorders, such as Alzheimer’s disease (AD), autism spectrum disorder (ASD), and stroke (Power et al., 2016; Kang et al., 2021), etc. For example, evidence from epidemiology has illustrated that exposures to PM_2.5_ in 2 years postnatal was linked with an increased risk for ASD in a population-based case-control study in Pennsylvania (AOR = 1.45, 95% CI = 1.01-2.08) (Talbott et al., 2015). It has been suggested that PM_2.5_ causes damage to the nervous system is because PM_2.5_ not only deposited in the lungs but also can enter the bloodstream, and cross the blood-brain barrier (BBB) (Shou et al., 2019). Autism spectrum disorder (ASD) is a neurodevelopmental disorder characterized by deficits in social and communication interaction, along with repetitive and stereotyped behaviors (First, 2013). The global prevalence of ASD was about 1%, with a higher prevalence of 1.5% in developed countries (Zeidan et al., 2022), and it usually starts in infancy and is predominantly in children, with a male-to-female ratio of about 4:1 (Lyall et al., 2017). The pathogenesis of ASD has not been fully understood, and its treatment is generally symptomatic to alleviate complications, with no specific treatment drugs available, which imposes a serious burden on families and society (Buescher et al., 2014). Studies have regarded ASD as the result of a combination of genetic and environmental factors (Tordjman et al., 2014), and epigenetic alterations have been found in individuals with ASD, such as DNA hypermethylation or hypomethylation (Wong et al., 2019), and non-coding RNA (ncRNA) expression alterations (Wu et al., 2016).

Transcriptome information represents the complex interactions among genome structure, dynamic gene expression homeostasis, and environmental signals, which enable the performance of transcriptome research to gain valuable information on complex genetic-epigenetic-environmental interactions (Ziats et al., 2015). Circular RNA (CircRNA) is a long-chain non-coding RNA in the transcriptome that is derived from precursor mRNA (Chuang et al., 2018). Unlike linear RNAs, circRNAs have a circular closed structure formed by covalent bonds. As a result, circRNAs are highly stable and conserved among different species and are considered as ideal biomarkers for some diseases (Rybak-Wolf et al., 2015). Currently, miRNA sponges act as one of the earliest and most important functions of circRNA. Since, circRNAs contain many miRNA binding sites that make circRNA combine with miRNA to regulate the expression of miRNA, and then affects the expression and function of mRNA (Chen, 2020).

As an exogenous environmental pollutant, the influence of PM_2.5_ on the nervous system has always been concerned. Existing studies from transcriptome sequencing have found that PM_2.5_ exposure can affect the expression of non-coding RNAs (Huang et al., 2017). What is more, sequencing of the hippocampus of epilepsy patients and cortical tissues of autistic patients also revealed many differentially expressed circRNAs (DECs) (Chen et al., 2020; Gray et al., 2020). Recently, a large number of differentially circRNAs were found in the brain tissue of ASD patients, and found that circARID1A can regulate genes implicated in ASD by functioning as a sponge of miR-6368, which indicates circRNAs have a key role in ASD (Liu et al., 2021). However, the systemic analysis of the relationship between PM_2.5_ and ASD from the perspective of transcriptome is still rarely. In this study, rats exposed to showed ASD-like phenotype, transcriptome sequencing was performed using the hippocampal tissues and differentially expressed circRNAs were analyzed. Kyoto Encyclopedia of Genes and Genomes (KEGG) and Gene Ontology (GO) analyses were conducted to reveal the possible biological pathways and functions of DECs. The circRNA-miRNA-mRNA regulatory network provided a theoretical and experimental basis for subsequent research. Overall, our findings could provide strong evidence and vital ideas that PM_2.5_ exposure triggers an autistic-like phenotype and also contributes to changes in circRNA expression.

## 2 Materials and methods

### 2.1 Animal experiments and PM_2.5_ exposure

Research and animal care procedures were approved by the Animal and Human Use in Research Committee of the Tianjin Institute of Environmental and Occupational Medicine (IACUC of AMMS-04-2020-002), and all animal experiments were performed in accordance with relevant guidelines and regulations. Pregnant Sprague-Dawley (PSD) rats were purchased from Beijing Vital River Laboratory Animal Technology Co., Ltd. (animal certificate number: SCGXK 2016-0006). To avoid uncertain sex-dependent differences, only male offspring rats were analyzed in this study. The litters were adjusted to 6 male pups per dam at postnatal day (PND) 1 and were randomly divided into control and exposure groups. Pups in the control group were exposed to filtered air, while pups in the exposure group were exposed to concentrated ambient PM_2.5_ by the PM enrichment system (HRH-PM186, Huironghe Technology, China) combined with whole-body inhalation exposure system (HRH-MNE3026, Huironghe Technology, China). The exposure period is from PND 1 to PND 21, during this period, pups were exposed simultaneously for 4 h/day and 7 days/week, and PM_2.5_ were concentrated approximately 8-fold the level in ambient outdoor air. And all animals were housed in standard cages under the conditions of 22°C ± 1°C, 50%–70% relative humidity, and 12 h light/dark cycle, with free access to food and water.

### 2.2 Marble burying test

Rats were placed in a cage (42 cm*24 cm*17 cm) evenly covered with 5 cm bedding and containing a total of 20 plexiglass beads in 5 rows of 4 each. The number of glass beads buried by the rats within 30 min was recorded.

### 2.3 Open field test (OFT)

The experiment was carried out in an open field box (100 cm*100 cm*50 cm), the bottom of which was divided equally into 25 small squares, the 9 in the middle of which were defined as the “central area” and the rest as the general area. A digital camera was placed 2 m above the chamber to record the behavior of the rats in the chamber for 5min, including the number of crossings of the central area, the total distance walked, and the number of bowel movements.

### 2.4 Novel object recognition (NOR) test

The experiment was conducted in three phases, the adaptation, familiarisation, and test phase, in an experimental plastic box (60 cm*40 cm*22 cm). Rats were placed in the test chamber and allowed to habituate for 5 min. During the familiarization stage, two identical objects (Greiner^®^ cell-culture flask filled with sand, 9.5 cm *2.5 cm *4.2 cm) were placed at equal distances from the wall and the rats were allowed to explore for 5 min. The testing phase was conducted after 30 min, one of the two identical objects was replaced with a similar one (Abcam^®^ Lego brick, 7.6 cm*1.9 cm*5.5 cm) and the rats were permitted to explore it for 5 min. A camera was suspended above the box to record the rats’ behavior. Clean and dried clear plastic boxes with 75% ethanol are required when completing a test.

### 2.5 Tissue collection and RNA extraction

Rats were killed *via* decapitation. Thereafter, their brains were dissected on ice and the hippocampal tissues were obtained. The TRIzol method was used to extract RNA from rat hippocampus; the RNA samples were strictly controlled. Agarose gel electrophoresis was performed to determine the integrity of the RNA and possible contamination; and NanoPhotometer^®^ spectrophotometer (IMPLEN, CA, United States of America) was used to detect RNA purity, the Agilent 2100 bioanalyzer was used to accurately detect the integrity of RNA (Agilent Technology, CA, United States).

### 2.6 Library construction

CircRNA sequencing required the removal of ribosomal RNA from total RNA to obtain lncRNAs. RNase R enzyme was then used to degrade linear RNA molecules to obtain circular RNA. The obtained circRNAs were randomly interrupted and a library was constructed according to a chain-specific method. First, the mRNA was enriched with Oligo (dT) magnetic beads, broken into short fragments, and used as a template. Six-base random primers (random hexamers) were used to synthesize one-strand cDNA, and then the RNA strand was digested in the cDNA hybrid with RNase H. The second strand was synthesized with DNA polymerase I and AMPure XP beads (Beckman Coulter, Beverly, United States) was used to purify the double-stranded cDNA. The purified double-stranded cDNA was then repaired, A-tailed, and connected to the sequencing adapters. The target fragments were recovered by agarose gel electrophoresis for PCR enrichment to obtain the final cDNA library.

### 2.7 Library quality and sequencing

After library construction, a Qubit21.0 Fluorometer was used for preliminary quantification, and the library concentration (1.5ng/uL) was detected. The Agilent 2100 bioanalyzer was then used to detect the quality of the library. When the insert size reached the expected value, qRT-PCR was performed to quantify the effective concentration of the library (the effective concentration of the library was higher than 2 nM) to ensure the quality of library. Illumina Novaseq6000 was used to sequence the transcriptome library after quantification. The PE150 (paired-end 150 bp) sequencing process was employed as the running program.

### 2.8 Illumina high-throughput sequencing

After qualified library inspection, different libraries were pooled according to the effective concentration and target offline data volume for Illumina sequencing. For synthesizing accompany Sequencing, herein, four fluorescently labeled dNTPs, DNA polymerase, and adaptor primers were added to the sequencing flow cell for amplification. When the complementary chain of each sequencing cluster was extended, the corresponding fluorescence could be released by adding a fluorescence-labeled dNTP. Then, the sequencer captured the fluorescent signal and converted the light signal into a sequencing peak through computer software; the sequence information of the fragment to be tested was finally obtained.

### 2.9 Identification and evaluation of circRNA

The CircRNA Identifier (CIRI) software was used to identify circRNA (Gao et al., 2015), scan the result files of BWA-MEM, and search for paired chiastic clipping (PCC), paired-end mapping (PEM) sites, and the GT-AG splicing signals. Finally, the sequence was re-aligned with junction sites using a dynamic programming algorithm to ensure the reliability of circRNA identification and to identify the circRNAs for subsequent analysis. However, owing to the particularity of circRNAs, accurately obtaining the read information of all circRNAs in the alignment is generally difficult, and is affected by linear RNA. The expression level of circRNA was estimated based on the number of reads (junction reads) that span the splice site of circular RNA, and the SRPBM (spliced reads per billion mapping) normalization method was used to quantify the expression of circular RNA. Based on the expression level, the Pearson correlation coefficient between each pair of samples was calculated using the cor function in R. The pheatmap function in R was also used to obtain the sample correlation cluster heat map.

### 2.10 Quality control and comparison of data

To ensure the quality of the analysis data, we used the cutadapt (V2.7) to filter the original sequence, trim the ends of reads with lower sequencing quality (sequencing quality value was less than 20), remove the reads that contain 10% of the N content, discard the adapters and small pieces that are less than 20 bp in length after quality pruning, and obtain the high-quality clean data. Data statistics were determined using fastqc (v 0.11.8), and subsequent analysis was performed with clean data. Clean reads were compared with the rat reference genome (http://asia.ensembl.org/Rattus_norvegicus/Info/Index) using the BWA-MEM algorithm in BWA (v. 0.7.17) to obtain mapped data. BWA-MEM can quickly and efficiently align reads with the genome and support the segmented alignment of the sequence to the genome (Jung and Han, 2022). After data comparison, RSeQC (V3.0.1) was used to evaluate the quality of the comparison results.

### 2.11 Differential expression analysis

The number of reads spanning a specific head-to-tail connection was determined as a measurement index for the circRNAs difference analysis. DESeq2 (R.V.1.24.0) was used for the differential gene expression analysis. The screening conditions for DECs were log2 fold change (log2FC) ≥2 and *p* < 0.05.

### 2.12 The function of circRNA predictions

CircRNAs are usually expressed by host genes. According to the Ensembl reference system (version GRCh37), we annotated the circRNAs using the splicing site and then predicted the function of circRNAs by the target genes. DAVID database (https://david.ncifcrf.gov/) was used to perform GO and KEGG enrichment analyses of the host genes of DECs. The hypergeometric test was used to determine the GO/KEGG term that was significantly enriched in the host gene of DECs compared with the background of the whole genome.

### 2.13 Validation of circRNA expression

The top ten circRNAs with significant differences were selected for qRT-PCR validation, including four upregulated and six downregulated circRNAs. Total RNA was extracted from the hippocampal tissues of rats using TRIzol reagent (Tiangen Biochemical Technology, China). Subsequently, RNA was reverse transcribed into cDNA using Revertaid M-MuLV enzyme, and qPCR was performed using Real-Time PCR System Step One Plus from ABI with the following reaction conditions: 30s pre-denaturation at 95°C, 95°C for 5 s, 60°C for 30 s, with 40 cycles; the melting curve analysis was performed between 60°C and 95°C. GAPDH was acted as the internal reference control and cDNA was the template. Two sets of primers, convergent and divergent primers (primers are listed in Supplementary Table S5) were designed. The relative expression levels in the target genes were calculated using the 2^−ΔΔct^ method. The PCR-amplified products were separated by 2% (w/v) agarose gels, and Sanger sequencing was used to test the authenticity of circRNAs.

### 2.14 Bioinformatic analysis of circRNA-miRNA-mRNA networks

We constructed the circRNA-miRNA-mRNA network based on the ceRNA hypothesis using Cytoscape3.9.1 to explore their interactions. The target miRNAs of DECs were analyzed using TargetScan (https://www.targetscan.org/vert-80/), and MiRWalk (http://mirwalk.umm.uni-heidelberg.de/) were also used to predict the genes targeted by miRNAs, and then the results were compared with the genes in SFARI database (https://www.sfari.org/) to obtain the intersection. Finally, the ceRNA network was depicted using the two validated circRNAs and the predicted miRNAs and mRNAs.

### 2.15 Statistical analysis

Statistical analysis was performed using both Social Sciences version 20 (SPSS 20) and GraphPad Prism 8. All data were presented as mean ± standard deviation (SD). Student’s t*-*test was used to assess the statistical significance, and a *p*-value <0.05 indicated the statistical significance.

## 3 Results

### 3.1 The influence of PM_2.5_ exposure on the behavior of rats

**FIGURE 1:**
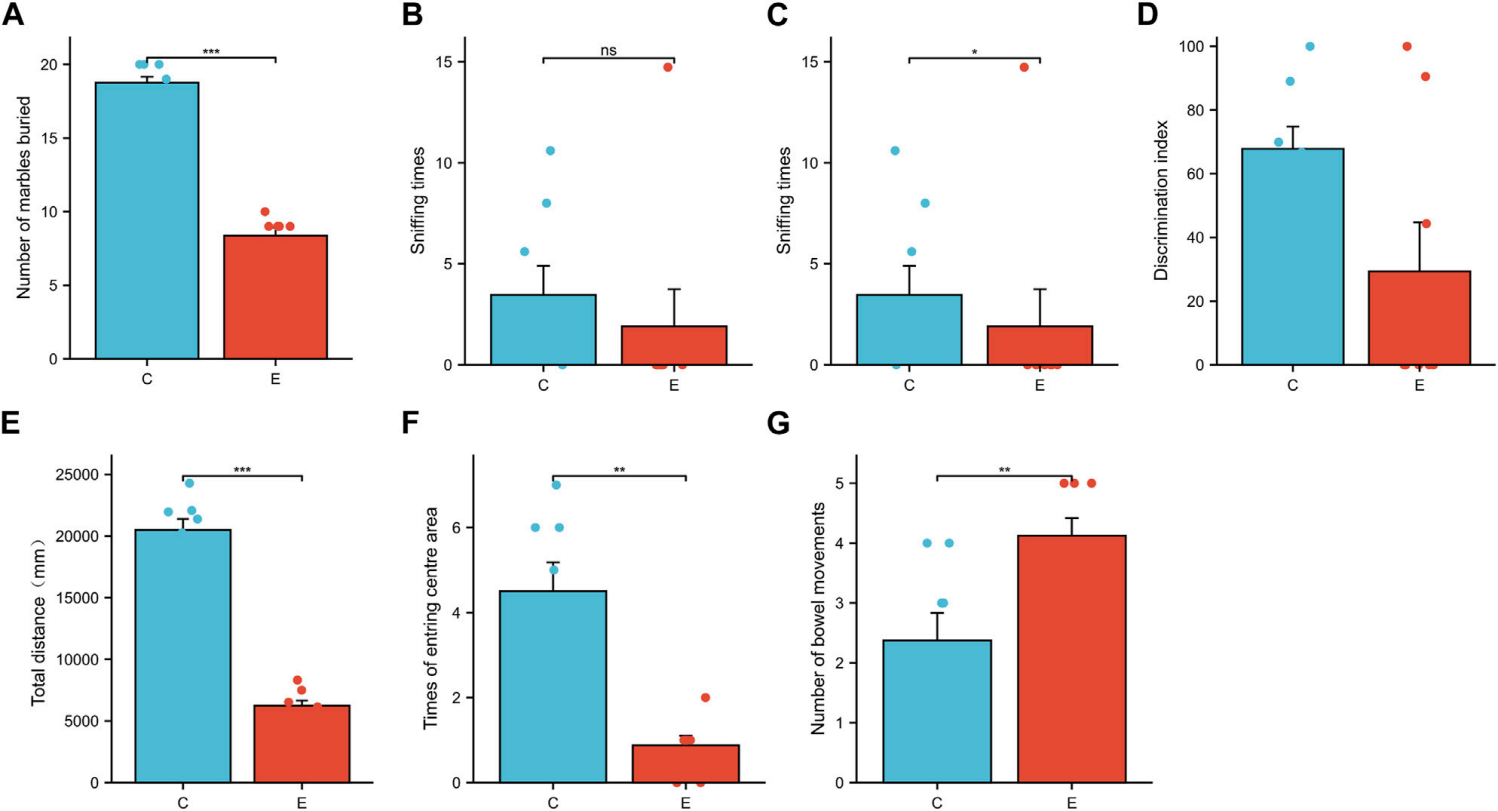
PM_2.5_-exposed rats exhibit anxiety, memory decline, and abnormal marble-burying behaviors. **(A)** The number of marbles buried in the marble burying test for control and PM_2.5_ exposure rats. **(B–D)** Novel object recognition (NOR) test. **(B)** Sniffing time for two identical objects. **(C)** Sniffing time for two similar objects. **(D)** Discrimination index = [(time for sniffing novel objects/total time for sniffing two objects)*100]. The experiment time is 5min, error bars represent the SEM. *p < 0.05,**p < 0.01,***p < 0.001 (n = 8 per group). **(E–G)** Open-filed test **(E)** The total distance that rats traveled in the experiment. **(F)** The number of times that rats entered the centre area. **(G)** The number of bowel movements of rats during the experiment.

Rats exposed to PM_2.5_ buried fewer marbles in the marble-burying test when compared to the control (Figure 1A), which is similar to the fear of novelty in children with ASD (Anckarsäter et al., 2006). From the novel object recognition test, we observed that the E groups spent less time sniffing new objects than the control groups (Figure 1C), and the discrimination index was also lowered in the E group, indicating that PM_2.5_ exposure undermined the learning memory capacity of rats. In the open field experiment, the total distance traveled and the central region crossed by rats exposed to PM_2.5_ were lower than in control groups (Figures 1E, F), while the defecations were more than that of controls (Figure 1G), indicating PM_2.5_ exposure made the rats more anxious and impaired their interest in exploring.

### 3.2 CircRNA expression in the hippocampus of rats

From our previous studies, behavioral tests and related indicators manifested that we already established the ASD-like model induced by PM_2.5_ exposure (Li et al., 2018). High-throughput sequencing enabled our research on circRNA expression profiles in the hippocampus of rats from the control and exposure groups. All the raw data were deposited into the NCBI Sequence Read Archive (SRA) database and could be accessed *via* accession number PRJNA813169. By sequencing the whole transcriptome, we obtained a total of 816 million original reads; 407 million belonged to the control group and 409 million original reads belonged to the exposure group (see Supplementary Material, Supplementary Table S1). After strict quality control of the original data, and the removal of sequences containing sequencing adapters, very short lengths, and markedly high N rates, 815 million clean reads were obtained, with 406 million clean reads in the control group and 409 million clean reads in the exposure group (**see** Supplementary Material, Supplementary Table S2). Subsequent analysis was performed using the quality-controlled clean data. The clean reads were compared with the rat reference genome (http://asia.ensembl.org/Rattus_norvegicus/Info/Index;VersionRnor6.0), which revealed a comparison efficiency between 99.494% and 99.718%, and the only comparison efficiency was also over 90% **(**see Supplementary Material, Supplementary Table S3).

**FIGURE 2:**
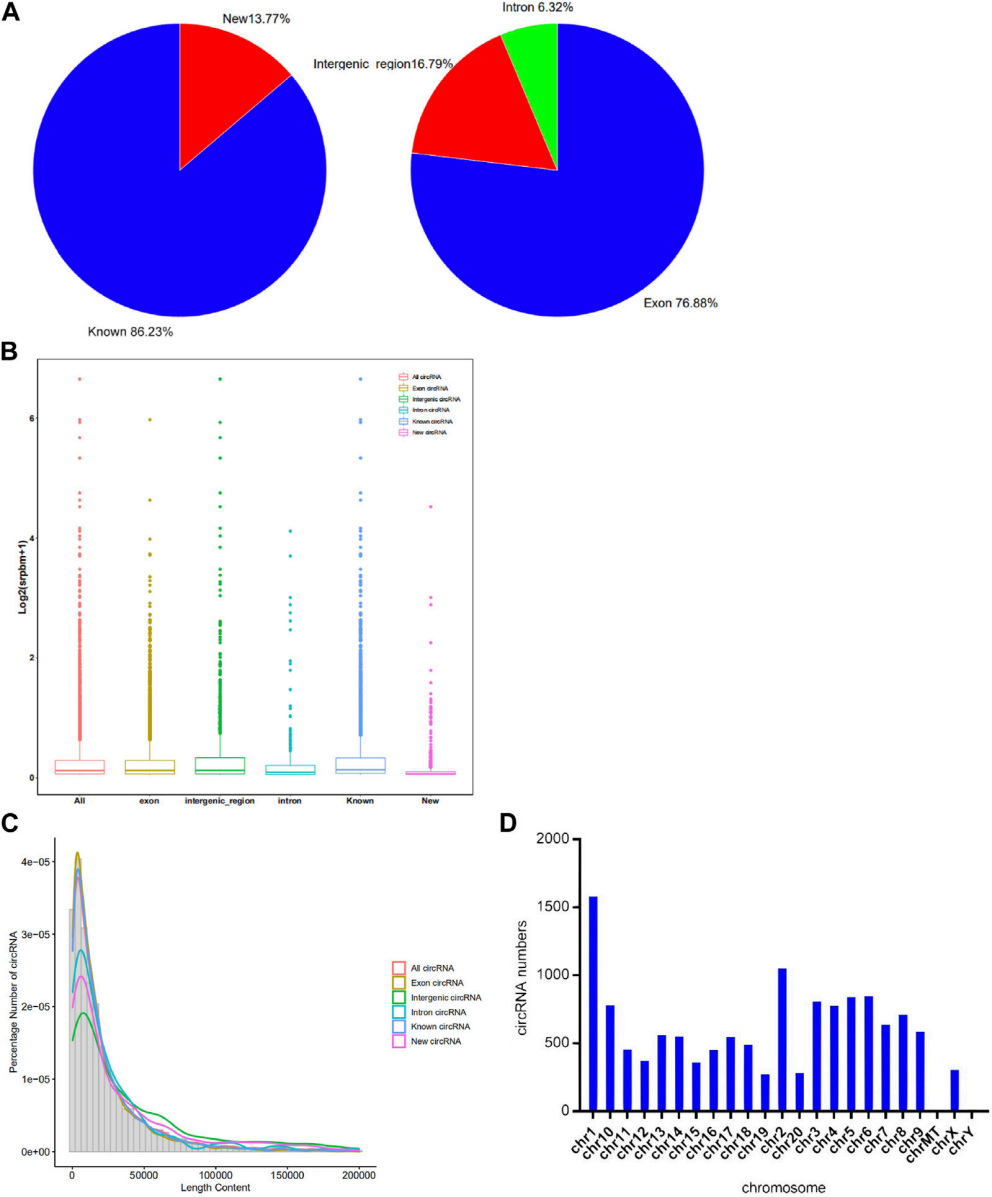
Characteristics of circRNA identified by Illumina Novaseq6000. **(A)** Bar graphs of circRNA expression profiles for different types of circRNAs. **(B)** Pie chart of total circRNA expression profiles. **(C)** The length distribution and frequency percentages of the sequences identified for different types of circRNAs. **(D)** The distribution of circRNA on each chromosome position.

A total of 7,770 circRNAs were identified in the control and exposure groups. Among these circRNAs, 86.23% were known and 13.77% were newly discovered. Interestingly, 5,973 circRNAs were classified as exons, 491 were classified as introns, and the remaining were intergenic circRNAs, which is presented in the Figure 2A. And from the Figure 2B, we found that the overall distribution of the circRNA data was relatively scattered. The expression of known circRNAs was higher than that of the unknown circRNAs. Exonic circRNA is the main type, higher than intron and intergenic circRNA. Different types of circRNAs were found to have similar peaks concentrated in the 10,417 bp regions. As shown in Figure 2C, the new and known circRNAs were similar in their length content, indicating that the new circRNA data are reliable. The circRNAs were distributed on almost all chromosomes in Figure 2D, with many circRNAs distributed on chromosomes 1 and 2, and few distributed on the Y chromosome and mitochondria.

### 3.3 Analysis of differentially expressed circRNAs (DECs)

**FIGURE 3:**
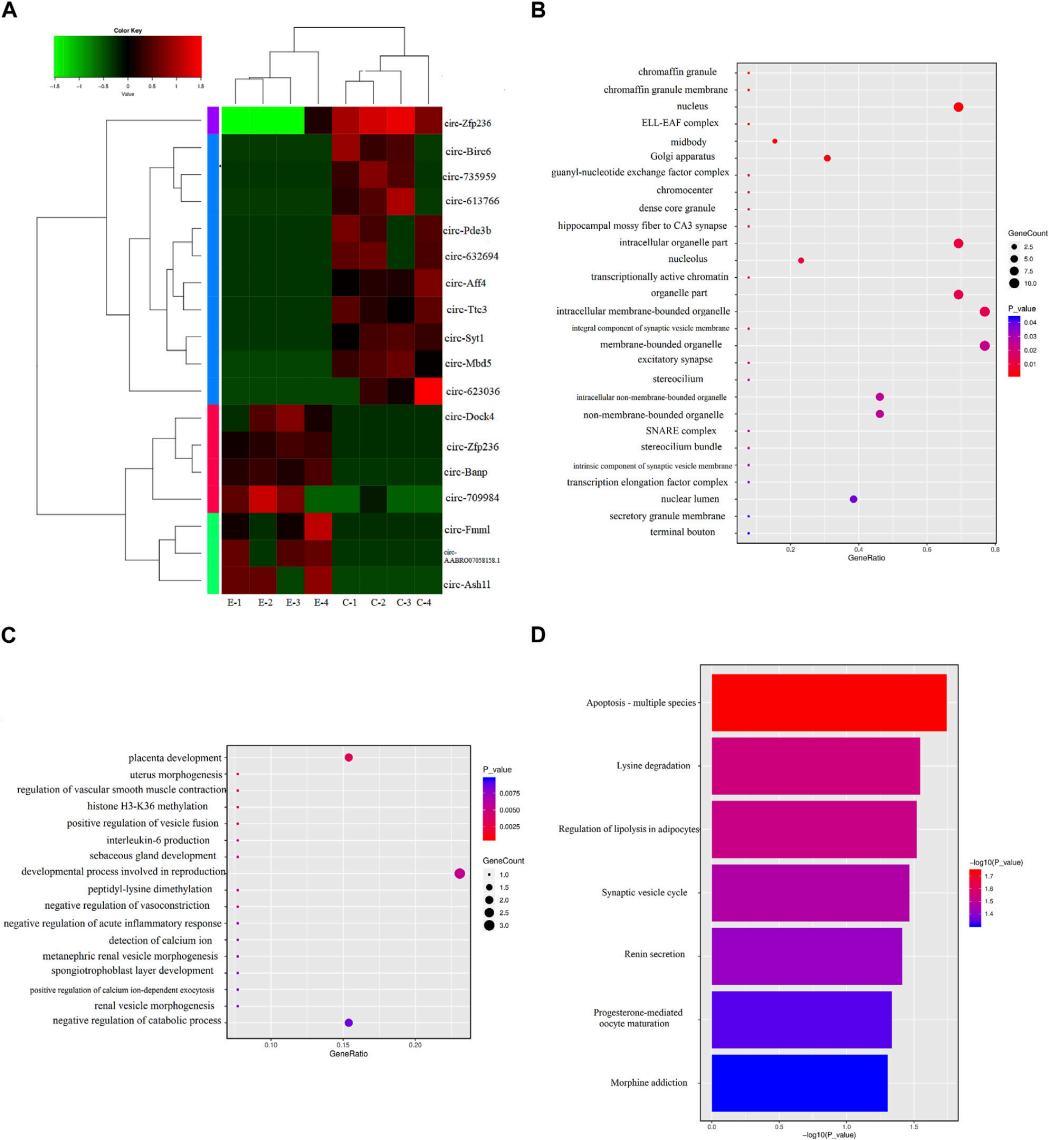
Differentially expressed circRNAs (DECs) between the control and exposure groups. **(A)** Heat-map of the expression levels of circRNAs in eight samples from the control (C1, C2, C3, and C4) and exposure (E1, E2, E3, and E4) groups. The expression levels of circRNAs were hierarchically clustered on the *y*-axis, and samples were hierarchically clustered on the *x*-axis. The colors of genes on the heat map correspond to the expression values normalized to circRNA means expression across all samples; green represented downregulated and red represented upregulated. **(B)** Kyoto Encyclopedia of Genes and Genomes (KEGG) pathway. **(C)** The cell component of Gene ontology for DECs. **(D)** The biological process of Gene ontology for DECs.

DECs are shown in Table 1, and the volcano and MA diagrams of DECs were shown in Figure 1 of the Supplementary Material. Hierarchical clustering revealed that the circRNAs were differentially expressed between the control and exposure groups. In fact, 18 DECs were found in the control and exposure groups (|log2FC|≥2 and *p* < 0.05), of which seven were upregulated and eleven were downregulated. We annotated the circRNAs with the protein-coding genes (host genes) by identifying the splicing junction site. To further study the function of circRNA, we used the DAVID database (https://david.ncifcrf.gov/) to analyze the function of circRNAs. Thereafter, GO and KEGG analyses were performed to determine the molecular pathway in which DECs were enriched. A *p*-value <0.05 was used as the threshold to select the most relevant biological function (Figure 3). Interestingly, among the “Biological Processes” category, DECs mainly involved in placental development, the processes of reproduction developmental and the catabolic (Figure 3D), indicating that PM_2.5_ exposure caused damage to the development of rats and DECs were enriched. Similarly, in “Cell Components” terms related to the synaptic vesicle membrane, excitatory synapse, hippocampal mossy fiber to CA3 synapse, and chromaffin granules were significantly enriched. KEGG pathway enrichment analysis revealed that DECs were mainly involved in lysine degradation, synaptic vesicle circulation, and apoptosis-related process.

**TABLE 1:** DECs between the control and exposure groups.

CircRNA-ID	Chr	CircRNA-type	Source-name	Log2FC	*p*-Value	Regulate
circ-613766	1	Intergenic-region	/	−6.237576542	0.020026132	down
circ-Pde3b	1	exon	Pde3b	−5.890356922	0.046502986	down
circ-Aff4	10	exon	Aff4	−5.851685336	0.032097596	down
circ-Ttc3	11	intron	Ttc3	−5.989959098	0.023336146	down
circ-632694	12	intergenic-region	/	−5.998592775	0.036488863	down
circ-709984	15	intron	AABR07018078.1	3.796005753	0.016358264	up
circ-735959	15	Intergenic-region	/	−5.944343722	0.037288804	down
circ-623036	16	Intergenic-region	/	−6.567092317	0.012204659	down
circ-Zfp236	18	exon	Zfp236	5.574301998	0.03809238	up
circ-Banp	19	exon	Banp	5.801291875	0.021077027	up
circ-Ash1l	2	exon	Ash1l	6.464885531	0.006892382	up
circ-Fmnl	3	intron	Fmn1	5.807381362	0.041805783	up
circ-Nelfcd	3	intron	Nelfcd	−3.133220526	0.01546644	down
circ-Mbd5	3	exon	Mbd5	−6.193117227	0.012512472	down
circ-Birc6	6	exon	Bic6	−6.135432219	0.026734859	down
circ-Dock4	6	exon	Dock4	5.75735286	0.042188916	up
circ-AABR07058158.1	7	exon	AABR07058158.1	6.009878318	0.024450566	up
circ-Syt1	7	exon	Syt1	−5.802128926	0.033095635	down

### 3.4 Validation of the differential expression levels of circRNAs

**FIGURE 4:**
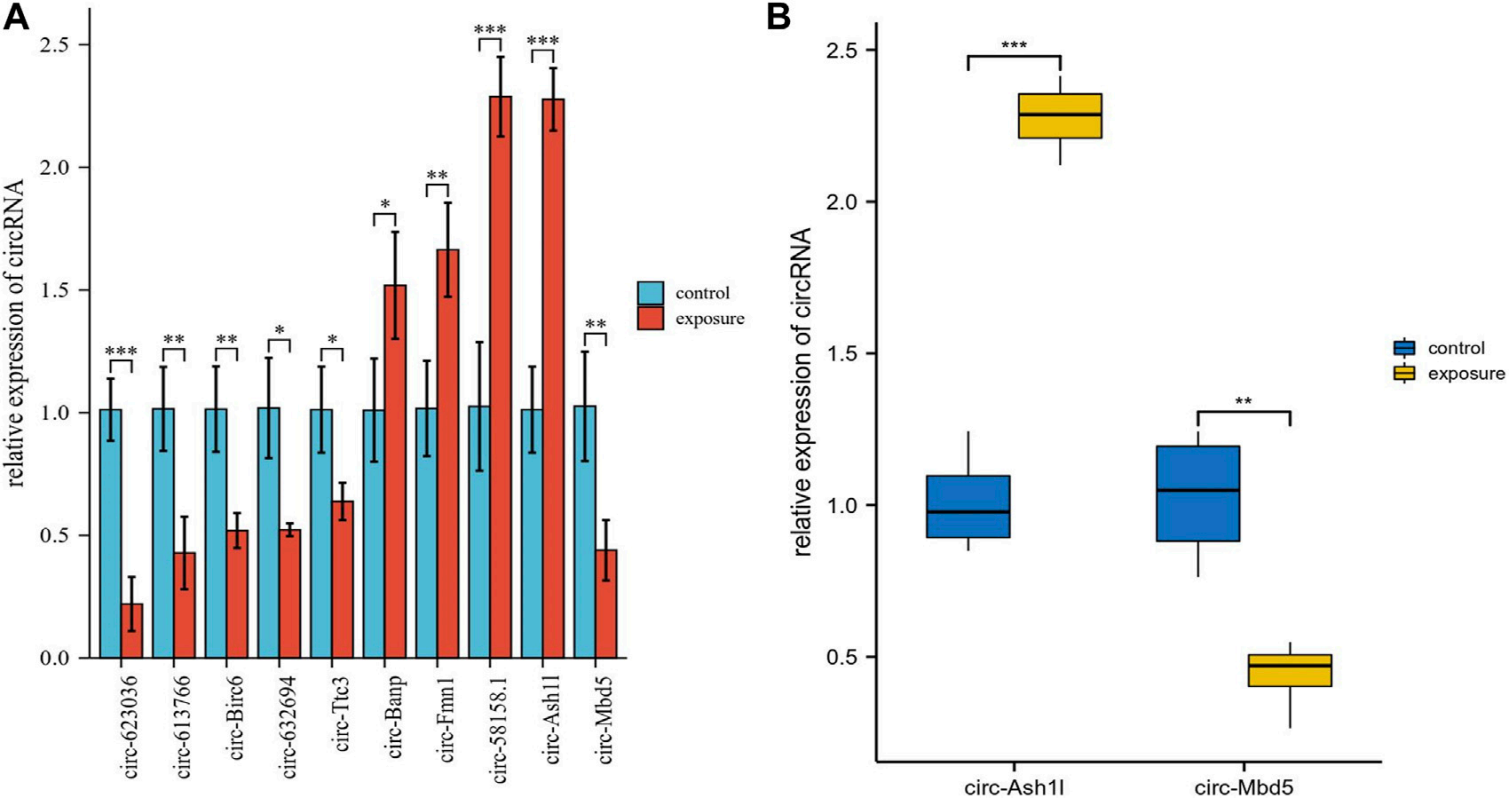
Validation of several differentially expressed circRNAs (DECs) from sequencing data using real-time PCR. **(A)** Ten circRNAs were selected for qRT-PCR validation and compared between the control and exposure groups. Data were presented as Mean ± standard deviation. Fold changes were calculated using the 2^−ΔΔCt^ method; *p < 0.05, **p < 0.01, ***p < 0.001. **(B)** Data distribution of the two most significant differences between *circ-Ash1l* and *circ-Mbd5.*

**FIGURE 5:**
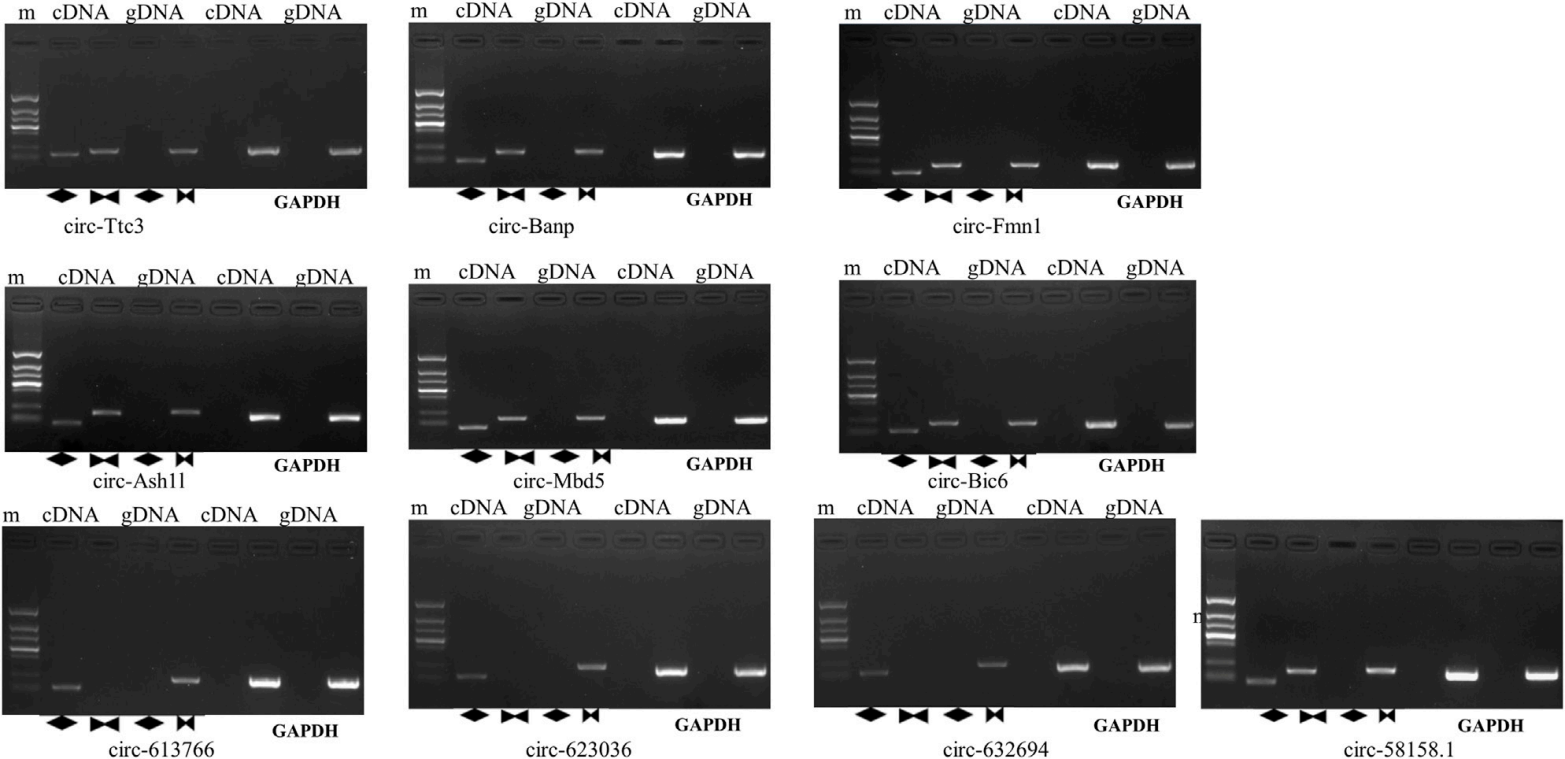
Evaluating the qRT-PCR products by agarose gel electrophoresis (AGE). M: maker, the amplified bands, from top to bottom, are 1500 bp, 900 bp, 700 bp, 500 bp, 400 bp, 200 bp, and 100 bp. In lane1,2,5,6 cDNA is the template, while in lanes 3,5,7,8, gDNA is used as the template. Lanes 1 to 4 amplify circRNA, and Lanes 5 to 8 amplify GAPDH. The diamonds in lanes 2 and 4 represent divergent prisms, the bowtie in lanes 3 and 5 represent convergent primers.

**FIGURE 6:**
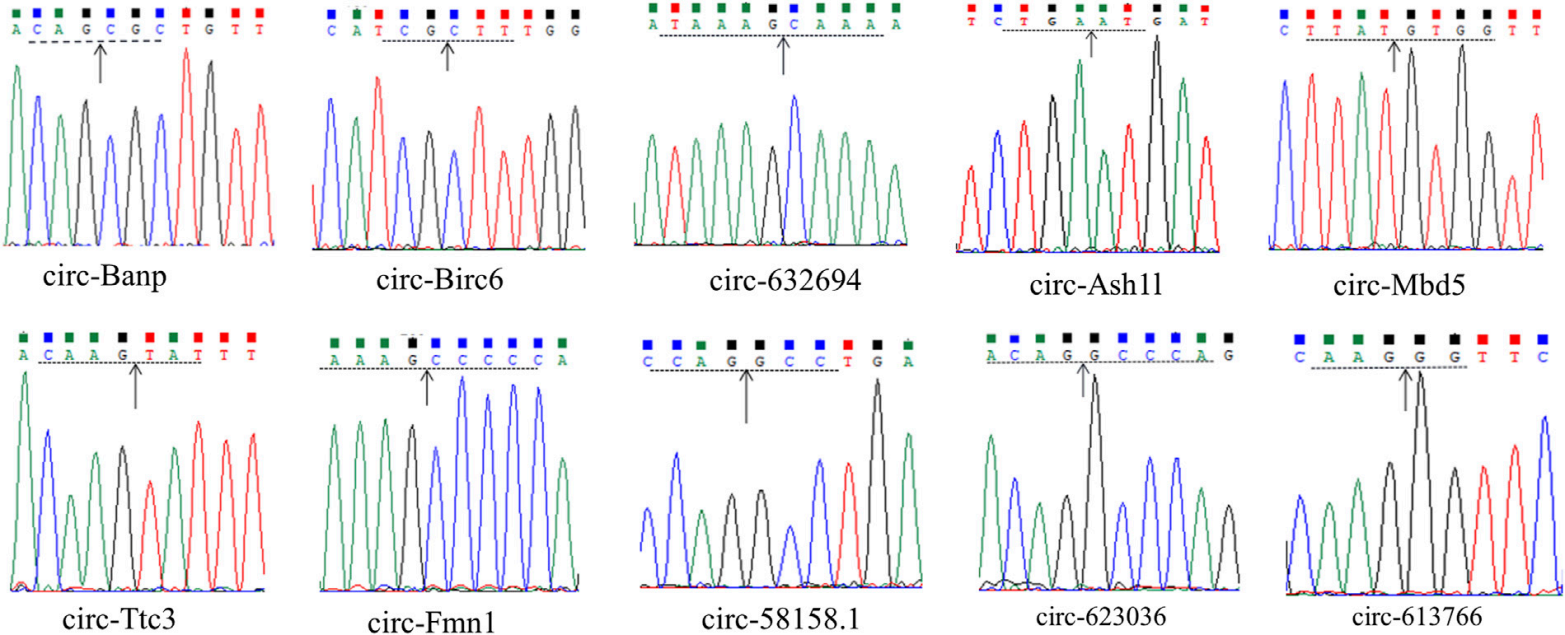
Sanger sequencing of the candidate circRNAs showed the back-splice junction. The arrows represented the “junction site” of circRNAs, and dashed lines indicate neighbouring base(s).

The ten circRNAs that were mostly dysregulated were selected for validation using real-time PCR. We designed two sets of primers, including divergent and convergent primers, and primer information for the circRNAs was provided in Supplementary Table S4. Based on the results in Figure 4, circ-Mbd5, circ-Birc6, circ-Ttc3, circ-613766, circ-632694, and circ-623036 were significantly downregulated, while circ-Banp, circ-Fmn1, circ- Ash1l, and circ-58158.1 were upregulated in the exposure group, compared with the control group. These results were consistent with the high-throughput data. Among them, the expression changes of circ-Mbd5 and circ- Ash1l were the most significant, therefore, we chose circ-Mbd5 and circ- Ash1l as the main circRNAs of subsequent research. As shown in Figure 5, agarose gel electrophoresis revealed that the ten candidate circRNAs could be explicitly amplified from cDNA by divergent primers, ultimately validating the circular structure of these circRNAs. The splicing sites of these circRNAs were also directly identified by Sanger sequencing (Figure 6). Thus, we confirmed that the selected candidates were indeed circRNAs.

### 3.5 Bioinformatic analysis of circRNA-miRNA-mRNA networks

**FIGURE 7:**
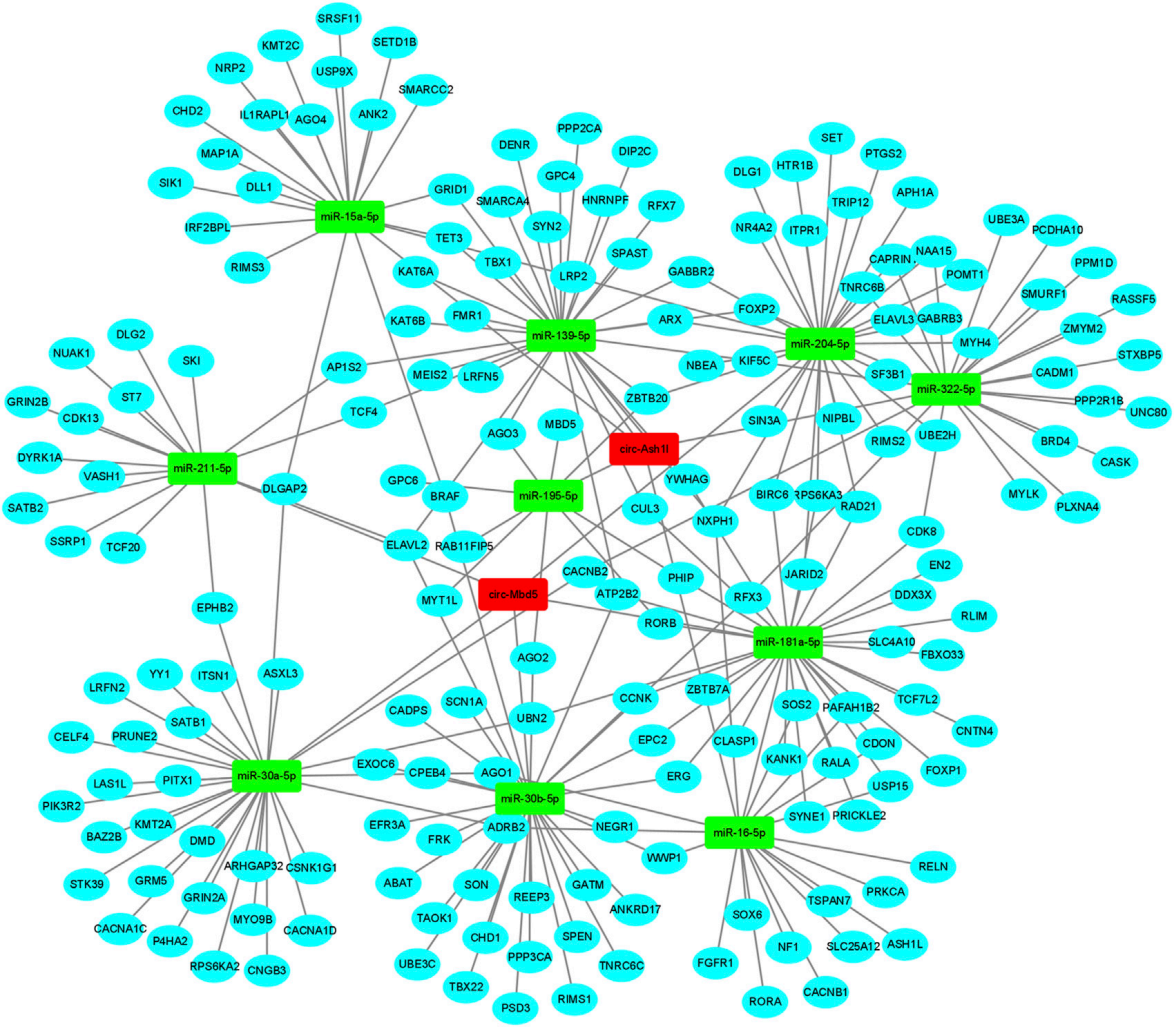
CircRNAs-miRNAs-mRNAs network. The ceRNA network was constructed with the two differentially expressed circRNAs (DECs). The red rectangle represented a circRNA, the green rectangle node represented miRNA and the blue round node represented mRNA.

The function of circRNA could be obtained through exploring the function of target miRNAs and mRNAs. To determine the function of *circ-Mbd5 and circ-Ash1l*, the TargetScan (http://www.targetscan.org/) and Miranda (http://www.microrna.org) databases were used to predict their target miRNAs by conserved seed-matching sequences. Firstly, we predicted the miRNAs of *circ-Mbd5 and circ-Ash1*l, and selected the top 5 miRNAs respectively; then, the target genes of the predicted miRNAs were compared and intersected with the risk genes of ASD in the SFARI database (https://www.sfari.org/), the 10 aforementioned miRNAs and the target genes for each circRNA were collected. The network, included 186 genes, many of which were highly associated with ASD such as ARX, CHD1, and FOXP_2_ (Figure 7). Therefore, this circRNA-miRNA-mRNA regulatory network could shed new light on the mechanism of ASD.

## 4 Discussion

Globally, PM_2.5_ levels are lower in regions such as Europe and the Americas, while higher in Southeast Asia and Africa (Yang et al., 2022). In northern China, PM_2.5_ levels are relatively high, especially in winter, and sometimes can exceed 100 μg/m^3^ daily average, which raises widespread concern (Shen et al., 2019). As a fine particulate matter, PM_2.5_ has a complex composition that includes both inorganic and organic substances, such as polycyclic aromatic hydrocarbons (PAHs), benzopyrene, nitrates, and metals (Yue et al., 2015). Owing to the small particle size, large surface area, strong activity, and slow sedimentation speed, they become a carrier for many chemical substances, bacteria, and viruses (He et al., 2017). As a result, PM_2.5_ is remarkably toxic and can pass through the respiratory tract and pulmonary capillaries to the alveoli, causing chronic obstructive pulmonary disease (Zou et al., 2021), asthma (Zhao et al., 2020), ischemic heart disease, and other diseases (Alexeeff et al., 2021). According to recent studies, PM_2.5_ is also associated with the occurrence of neurological diseases (Fu et al., 2019), such as Alzheimer’s disease (AD) and depression (Thiankhaw et al., 2022), the reason is that they can penetrate the cerebral cortex by damaging the tight junctions of the blood-brain barrier (BBB) (Shou et al., 2019; Kang et al., 2021). By assessing PM_2.5_ in this study, it was found to contain a variety of heavy metals and PAHs, such as manganese, cadmium, copper, zinc, and lead, with zinc accounting for the highest proportion. Thirteen species of PAHs were detected, with anthracene accounting for the highest content. During the study of particulate matter, whether PM_2.5_ itself or its components cause a series of changes cannot be deduced, which is an inevitable limitation. In the future, our research group will conduct further in-depth studies about PM_2.5_.

Epigenetics has been found to play an important role in the mechanism of neurological diseases. In the pathogenesis of ASD, except for genetic reasons, environmental factors also play important role in it (Waye and Cheng, 2018), and there have already caused many discussions about the impact of PM_2.5_ as a major air pollutant on ASD. Epidemiology suggested significant association between PM_2.5_ exposure and ASD, for example, Rahman conducted a population-based retrospective cohort study from the United States and found that prenatal exposure to PM_2.5_ was associated with ASD, and the association was stronger in boys (Rahman et al., 2022). A study from Shanghai of a case-control study concluded that the first 3 years of life exposure to particulate matter air pollution were associated with an increased risk of ASD (Chen et al., 2018). The above studies suggested that prenatal and postnatal PM_2.5_ exposure both had an effect on ASD. In addition, pregnant mice exposed to PM_2.5_ caused behavioral deficits in adult offspring early in neurodevelopment (Church et al., 2018), and from our previous experiment, ASD-like social interaction impairment and repetitive stereotyped behaviors were also observed in the SD rats, and levels of pro-inflammatory cytokines IL-1β, IL-6, and TNF-α were increased, and the expression of Shank3 was abnormal. (Li et al., 2018), which is the highly risk gene in ASD (Tzanoulinou et al., 2022). Taken together, epidemiological and animal evidence suggested that PM_2.5_ could be associated with ASD.

In view of the current diagnosis of ASD is mainly based on behavioral characteristics according to Autism Diagnostic Interview (ADI) and Autism Diagnostic Observation Schedule, Second Edition (ADOS-2) (Lord et al., 1994; Lord et al., 2013) or the diagnostic criteria of *Diagnostic and Statistical Manual of Mental Disorders, 5th ed*. (*DSM-V*) (First, 2013). Marble burying test, which was used to detect repetitive stereotyped behaviour in the animals, our experiment did not observe increased beads in the E groups compared with the control, which may be due to the rats deliberately avoiding exposure to new things (Anckarsäter et al., 2006). The number of bowel movements in the open-field experiment reflects the anxiety level of the rats in a strange environment, with more bowel movements representing a higher level of anxiety. The total distance travelled and the number of times the rat crossed the central area indicated autonomous and exploratory behaviour (Denenberg, 1969; Walsh and Cummins, 1976). Furthermore, autopsy reports allow direct studying and confirming the pathological changes in the brains of ASD patients, glial cells and astrocytes expression levels, oxidative stress levels, and the expression levels of non-coding RNA (ncRNA) were changed (Fetit et al., 2021). In our ASD model, the expression of GFAP and Iba1 was also significantly increased, and oxidative stress biomarkers were also significantly changed (Li et al., 2018). In addition, many differentially expressed ncRNAs were concentrated in our ASD model, which indicated that our PM2.5-induced ASD model was very ideal and successful in some aspects. It is known that a recent study in Taiwan showed that researchers identified many DECs in post-mortem brain samples from ASD patients and controls (Chen et al., 2020). CircRNAs are divided into various types according to the different splicing sites, which can be derived from exons, introns, and pre-tRNA (Chen, 2020). Approximately 86% of circRNAs in our study were exon circRNAs, which is consistent with Chen’s study, and most exonic circRNAs were distributed in the cytoplasm and were stably expressed in cells (Salzman, 2016). CircRNAs possess the circular covalently closed structure, they are not easily degraded by RNase and are therefore more stable than linear RNA. Moreover, circRNAs are stably and highly expressed in a variety of human tissues, especially the mammalian brain (You et al., 2015) and neuronal tissues (Chen and Schuman, 2016). CircRNAs are upregulated during neurogenesis, indicating that they are genuinely involved in neuronal phenotypes (Shao et al., 2021). For example, CiRS-7 is a widely studied circRNA that contains numerous anti-miR-7 binding sites that can sequester the miR-7 and affect its interaction with target gene mRNAs. In fact, CIRS-7 plays a role in various diseases, and is expected to become a new target for disease treatment (Zhao et al., 2016). Besides, evidence from animal experiments suggested that the overexpression of CircDLGAP4 could alleviate the neurological deficits and infarct size in a mouse stroke model, implying new insight into the treatment of stroke (Bai et al., 2018). It can be seen that circRNAs play an important role in neurological diseases, and our study of the effects of PM_2.5_ exposure on circRNAs can provide valuable clues for disease treatment.

CircRNAs play a variety of important biological roles, such as miRNA sponge, translation, and regulation of mRNA expression through RNA binding protein (RBP), etc. (Chen, 2020). We annotated the host gene of circRNA and performed enrichment analysis, indicating DECs mainly concentrated in the process of reproduction and placental development, and regulate catabolism. We inferred exposure to air pollution would affect the expression of placenta-imprinted genes (Kingsley et al., 2017), Kaur’s recent study also found that PM_2.5_ exposure altered the expression of placental genes related to lipid and glucose metabolism (Kaur et al., 2022), Deyssenroth’s research is similar (Deyssenroth et al., 2021). CircRNA expression was dysregulated in postnatal pups after PM_2.5_ exposure in our study, suggesting that early birth PM_2.5_ exposure may also alter placenta-related gene expression. As for damaging reproduction development, it may be because PM_2.5_ exposure can adversely affect spermatogenesis and the reproductive system by reducing testicular and follicle-stimulating hormone levels, reducing sperm production in the epididymis, causing misalignment of germ cells and altering spermatogenic tubules (Qiu et al., 2018). CircRNAs contain multiple miRNA-binding sites and miRNAs may target multiple mRNAs, and circRNA-Mbd5 and circ-Ash1l can regulate the expression of miRNAs in Figure 6. In the study, circular structures of circ-Mbd5 and circ-Ash1l were validated by agarose gel electrophoresis and Sanger sequencing, which indicated that circRNAs were genuine circRNAs, and we next will conduct relevant experiments to verify the regulatory relationship in the network.

## 5 Conclusion

We conducted animal behavioural experiments to investigate the effects of PM_2.5_ exposure on rats in this study, and identified many circRNAs based on high-throughput sequencing of the hippocampus of ASD-like rats and further explored the functions of these circRNAs by combining KEGG, and GO analysis. Some biological processes in which many circRNAs are involved are affected, implying PM_2.5_ disturb it. In subsequent studies, we will conduct further research on the ceRNA regulatory network of circ-Mbd5 and circ-Ash1l with related miRNAs and mRNAs, as well as the influence of PM_2.5_ exposure on the expression of circ-Mbd5 and circ-Ashl1.

## Data Availability

The data presented in the study are deposited in the NCBI Sequence Read Archive (SRA) repository, accession number is PRJNA813169 (https://www.ncbi.nlm.nih.gov/sra/?term=PRJNA813169).
