# Supplementary material for: Identification and characterization of circular RNA in the model of autism spectrum disorder from PM2.5 exposure
Source: Front Genet. 2023 May 9;14:970465. doi: 10.3389/fgene.2023.970465 (PMC10203163; doi:10.3389/fgene.2023.970465)
Supplement: Supplementary file 1 [file DataSheet1.PDF]

Supplementary Material

1.1 Supplementary Tables 1

Table S1 Raw reads of control and exposure samples

Sample	Total_Reads number	Total_Bases number	Q30%	GC%
C-1	112710118	17019227818	93.18	45.44
C-2	88459168	13357334368	92.93	45.72
C-3	108001038	16308156738	92.5	46.26
C-4	97375414	14703687514	92.85	46.15
E-1	104990024	15853493624	92.62	46.23
E-2	109174120	16485292120	92.64	46.47
E-3	105510978	15932157678	92.9	45.91
E-4	89695796	13544065196	90.78	46.27

1.2 Supplementary Table 2

Table S2 Clean reads of control and exposure samples

Sample	Total_Reads number	Total_Bases number	Q30%	GC%
C-1	112614296	16252753024	93.56	45.1
C-2	88386178	12692248181	93.45	45.34
C-3	107900932	15656533579	92.93	45.99
C-4	97291862	14077686501	93.25	45.84
E-1	104880146	15196233531	93.11	45.93
E-2	109057044	15864349872	93.08	46.19
E-3	105413346	15304478736	93.26	45.64
E-4	89617790	13087025311	91.18	46.02

1.3 Supplementary Table 3

Table S3 mapped reads of control and exposure samples

sample	total_reads	total_map	positive_map	negative_map
C-1	112614296	112296297(99.718%)	51341232(45.590%)	51352263(45.600%)
C-2	88386178	88111510(99.689%)	40082034(45.349%)	40094710(45.363%)
C-3	107900932	107513909(99.641%)	48959545(45.375%)	48970140(45.384%)
C-4	97291862	96981620(99.681%)	44333009(45.567%)	44343170(45.577%)
E-1	104880146	104326684(99.472%)	47620442(45.405%)	47627307(45.411%)
E-2	109057044	108631033(99.609%)	49437519(45.332%)	49448822(45.342%)
E-3	105413346	105027401(99.634%)	48002814(45.538%)	48012151(45.547%)
E-4	89617790	89164246(99.494%)	40662891(45.374%)	40671949(45.384%)

1.4. Supplementary Table 4

Table S4: Primers designed for qPCR validation of candidate circRNAs

CircRNA ID		Primer	Size（bp）
circ-623036 Divergent Primers	F	CAGAGAACTCCGTAGAGCACCAC	105
	R	CAGTTCATGTCTGACACACTGGATC	
circ-623036 Convergent Primers	F	GCTGTGTAAATGGAGGGGGC	153
	R	GTGGTGTCAGGAAGAACAAAGGG	
circ-613766 Divergent Primers	F	GAGGACAATGTGAAATGGAAAAGG	111
	R	CAGAACTTAGTGGAGAAAAAGCACCT	
circ-613766 Convergent Primers	F	GATTCTAACACTGGAGATTACAACC	191
	R	GATTTTGTGAAAGGGCATAAGAC	
circ-Mbd5 Divergent Primers	F	CACAGTGTTCATCAAAGGAGCAT	62
	R	GAGGACGGCAGGCTTGAAC	
circ-Mbd5 Convergent Primers	F	TGTTACCACCAAGAAACTGTCAAGG	155
	R	CCGACTCTGACGAGGTCTGTTTC	
circ-Birc6 *Divergent Primers*	F	ATCTGGAATGCTCTTCGCTGTC	109
	R	CCAGGATACATTTGTTTCTGTCATTT	
circ-Birc6 *Convergent Primers*	F	CAGTCTCACCTCTCCAGATTCCG	162
	R	CGCAGTCCGACCAACAAATG	
circ-632694 Divergent Primers	F	GAAGTCACTCAAGATAATGAATGCC	115
	R	CCAGGCTTGTGTATTAGTGTTGTC	
12:587083|632694 -Convergent Primers	F	AACATAGGGTTGACTTGTCTTAGG	182
	R	ATAGTCTGCTCTGGTGAAGAATGT	
11:34600704|34624408 Divergent Primers	F	CTCGGCTGGAGAAAGTATAAATG	127
	R	AGTTAGTGTTCATTTGTTTGACTTCTC	
11:34600704|34624408 Convergent Primers	F	AGTATGACATCTGCAATATATGGT	151
	R	ATGGCTTCTACACAGGGGT	
circ-AABR07058158.1 Divergent Primers	F	GGGAGGGTGAGCATTGGTTG	75
	R	TCAGCAGGGTGGAGTTAAGAAAATC	
circ-AABR07058158.1 Convergent Primers	F	GCCTGCCTAGAGATGTTACAAGTG	142
	R	TAGAGGGAGCCCGAGAGAGC	
circ-Banp Divergent Primers	F	GGAAGTTCCTTTTTGTTCTGTGC	94
	R	ATGGTATTCTGCCGACGCC	
circ-Banp Convergent Primers	F	CCTGGCTGGGTGATGAGAATA	141
	R	CCTCACGGTGGAACAGGTAGT	
circ-Fmnl Divergent Primers	F	ACTCAACAAAAGAAAAAACCGCTG	94
	R	TGGCTGAGGCTAACCTACGACAT	
circ-Fmnl Convergent Primers	F	GGGGAGAATGTGAAGAGATGAGA	163
	R	TTCTTGGGCACTATCTGGCTG	
circ-Ash1l Divergent Primers	F	GTACACCCGATGGAATGGC	78
	R	GTTAGGGGAACACTCAGCAAA	
circ-Ash1l Convergent Primers	F	CCCTGTGGTGAGCAGTGCTGTAA	159
	R	GCTGACAACTTCCCCCAGGTATT	
GAPDH -Convergent Primers	F	TTCAGCTCTGGGATGACCTT	129
	R	TGCCACTCAGAAGACTGTGG	
GAPDH - Divergent Primers	F	CCTTCCGTGTTCCTACCCC	N/A
	R	GGGATGATGTTCTGGGCTG	

2. **Supplementary Figure I**

Volcano diagram and MA diagram of differentially expressed circRNA

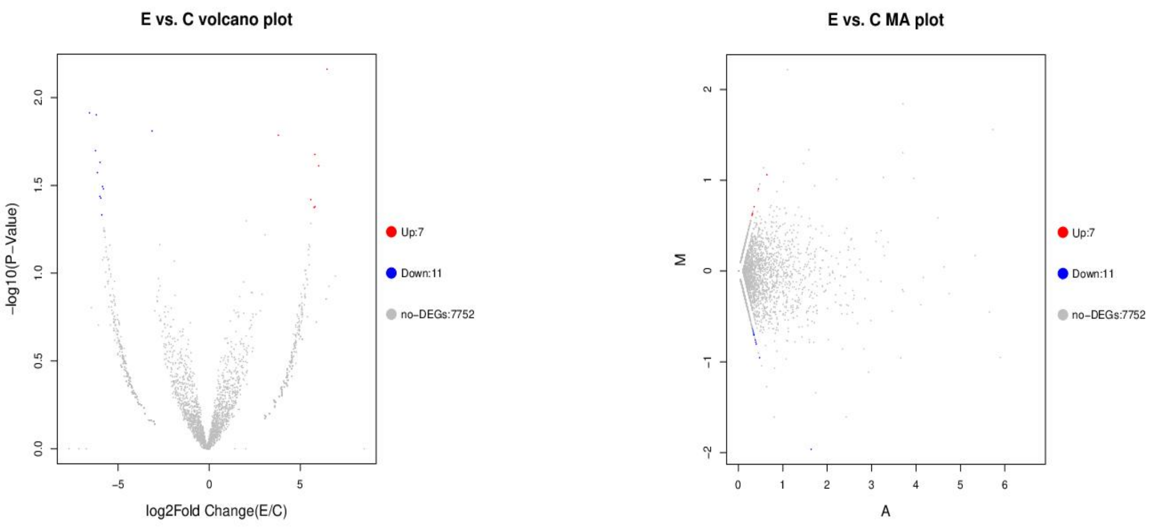

Supplementary Figure 1 : Each point represents a circRNA. The horizontal coordinate is the multiple of circRNA expression difference between the two groups. The red point is the differentially up-regulated circRNA, and the blue point is the down-regulated circRNA.
